# Supplementary material for: Intrinsic bias estimation for improved analysis of bulk and single-cell chromatin accessibility profiles using SELMA
Source: Nat Commun. 2022 Sep 21;13:5533. doi: 10.1038/s41467-022-33194-z (PMC9492688; doi:10.1038/s41467-022-33194-z)
Supplement: Supplementary file 1 — Supplementary Information [file 41467_2022_33194_MOESM1_ESM.pdf]

**Supplementary Information**

# Intrinsic bias estimation for improved analysis of bulk and single-cell chromatin accessibility profiles using SELMA

Shengen Shawn Hu, Lin Liu, Qi Li, Wenjing Ma, Michael J. Guertin, Clifford A. Meyer, Ke Deng, Tingting Zhang, and Chongzhi Zang

This Supplementary Information file includes Supplementary Figures 1–12.

## Supplementary Figure 1

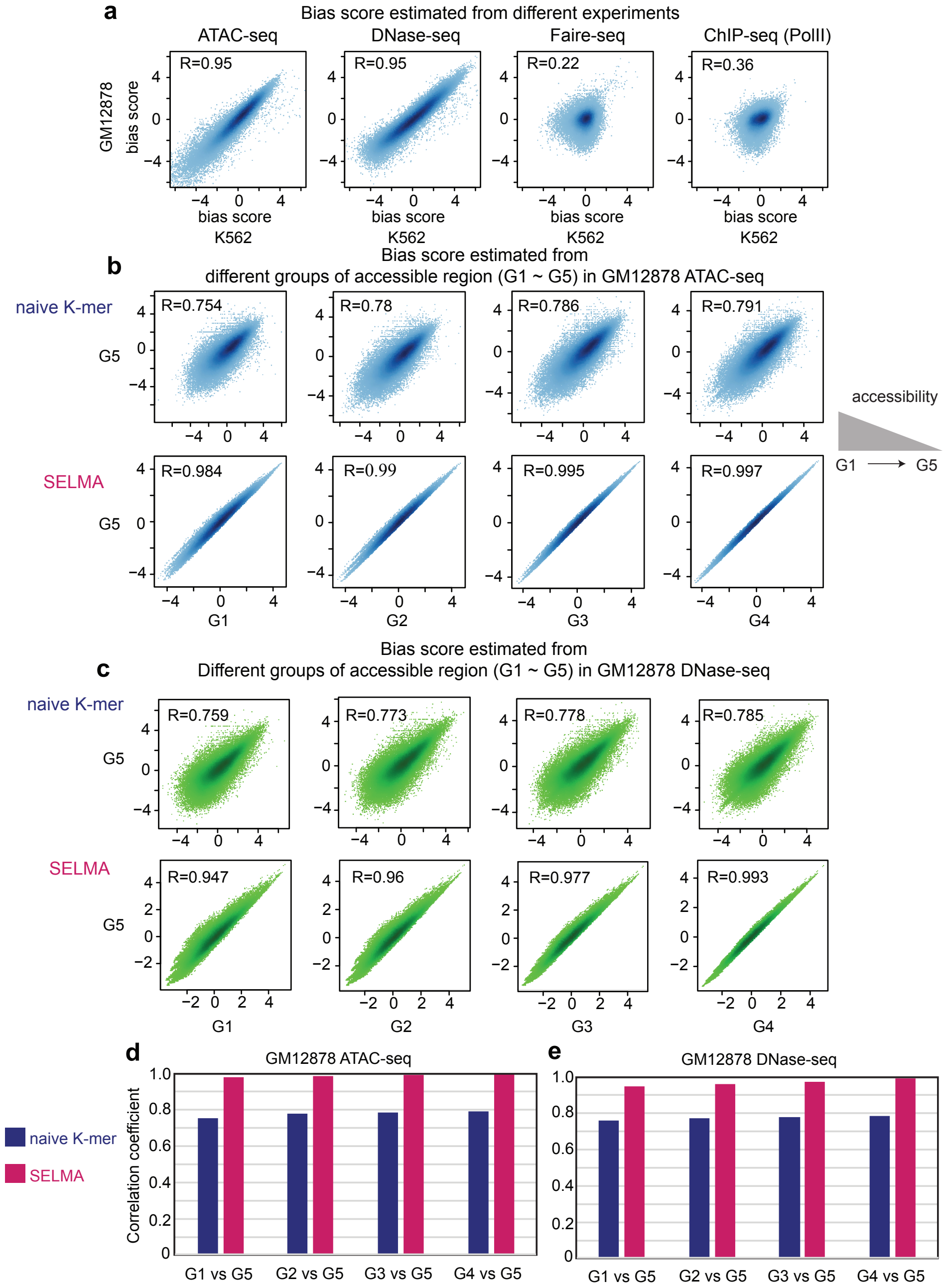


**Supplementary Figure 1. Improvement of bias estimation of the SELMA model.** (**a**) Comparison of intrinsic cleavage bias estimated in different data types. Each dot in a scatter plot represents an 8-mer, with its estimated bias score from K562 and GM12878 cell lines represented in x and y axes, respectively. Different plots represent bias estimated using data from different experiments (ATAC-seq, DNase-seq, Faire-seq, and ChIP-seq). R represents the Pearson correlation coefficient. (**b**) Comparison of Tn5 intrinsic cleavage bias estimated from different groups of fragments. The top panels are for the 8-mer bias estimated using the naïve k-mer model, and the bottom panels are using the SELMA model. DNA fragments are separated based on the region's chromatin accessibility, illustrated in the schematic on the right, and labeled at the axes. Fragments located in the top 20% chromatin accessible regions with the highest accessibility are assigned as group1 (G1). Then fragments located in the top 20%~40%, 40%~60%, 60%~80%, and bottom 20% accessible regions are assigned as G2, G3, G4, and G5, respectively. (**c**) Similar to (b) but for DNase-seq bias score. (**d, e**) Barplots comparing the Pearson correlation coefficients (R) calculated in (**b**, **c**), respectively. The blue bars are for the naïve k-mer model, and the red bars are for the SELMA model.

## Supplementary Figure 2

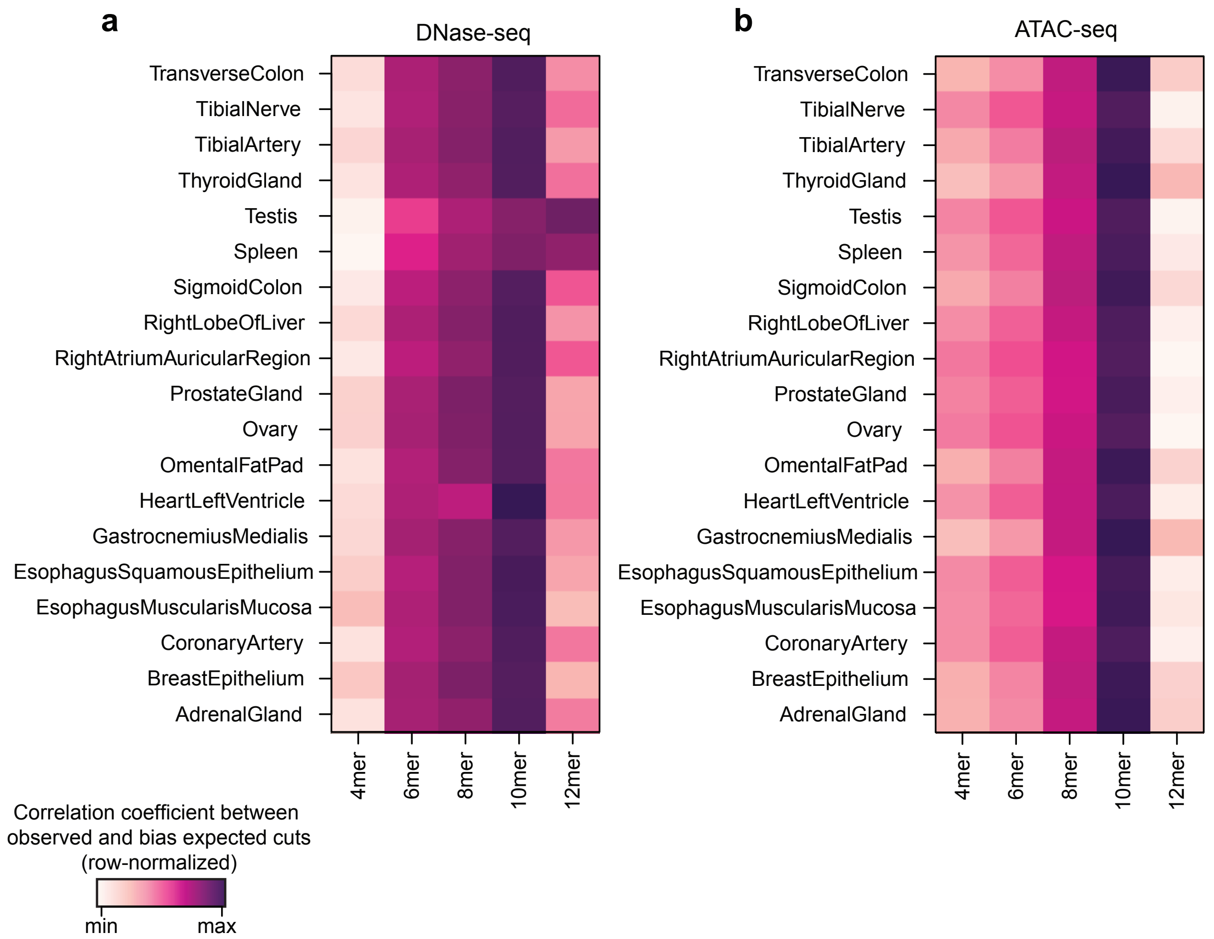


**Supplementary Figure 2. Intrinsic cleavage bias estimation accuracy with different k-mers.** Intrinsic cleavage bias estimation accuracy is measured by the correlation between genome-wide observed (OBS) and bias-expected (EXP) cleavages with different k-mers for ENCODE DNase-seq data (**a**) and ENCODE ATAC-seq data (**b**), respectively. Different rows represent data from different tissues. The Pearson correlation coefficients (R) in the same cell type (each row) are Z-normalized to compare the relative performance of SELMA models with different k-mers. The higher the normalized score, the better performance the model has.

## Supplementary Figure 3

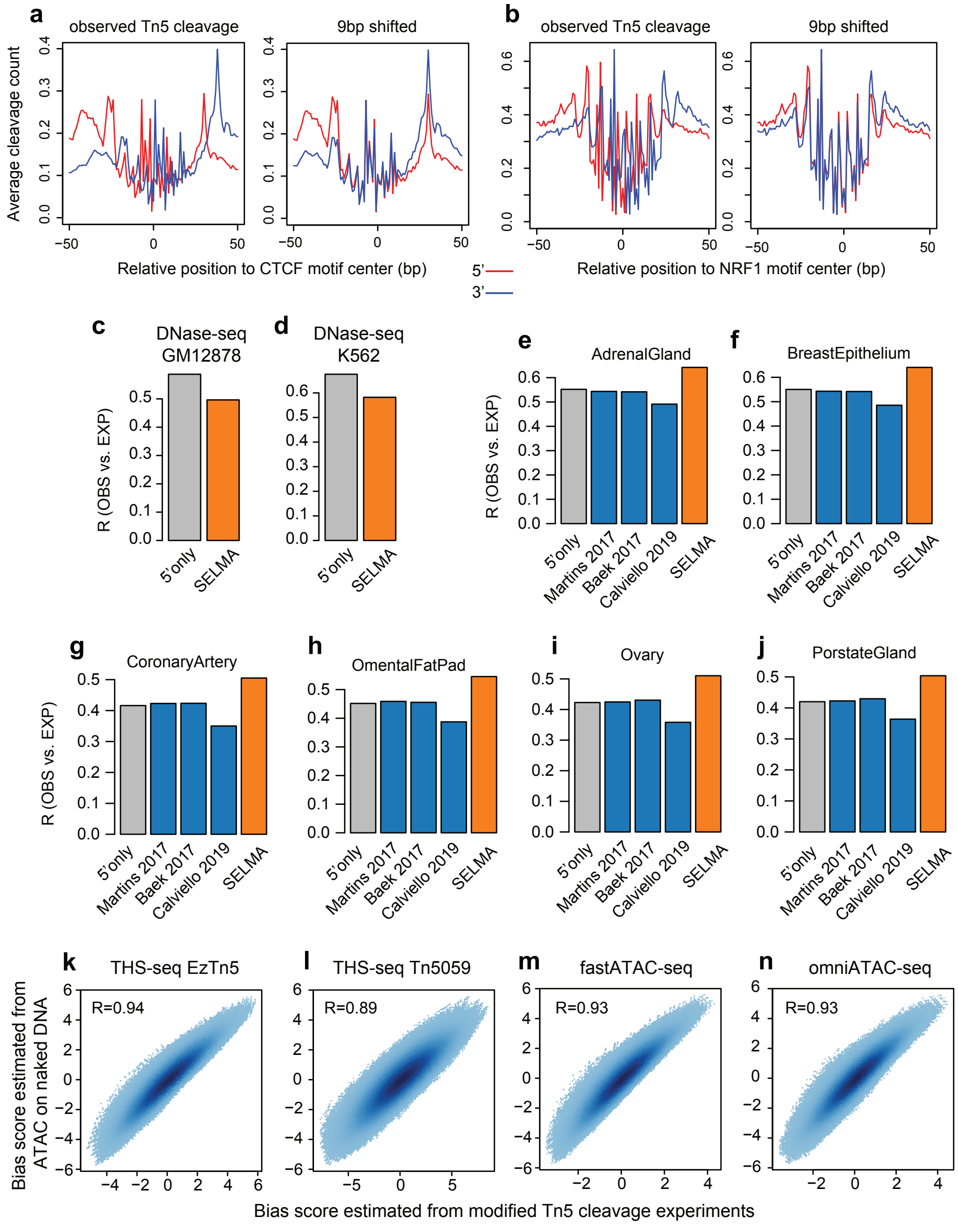


**Supplementary Figure 3. SELMA consideration of dimeric Tn5 cleavages for ATAC-seq.** (**a**) The composite profile of the ATAC-seq cleavage pattern around the CTCF motif sites. +/- strand reads are separated and plotted in red and blue lines, respectively. The left panel is for unmodified observed Tn5 cleavage. In the right panel, the - strand cleavage pattern are shifted 9bp towards upstream. (**b**) similar to (**a**) but for the NRF1 motif sites. A perfect match of + and – strand profiles can be observed for the 9bp shift. (**c, d**) Comparison of DNase-seq intrinsic cleavage bias estimation accuracy measured by Pearson correlation coefficient (R) between genome-wide observed and bias-expected cleavages for human GM12878 (**c**) and K562 (**d**) cell lines. Different bars represent different estimation approaches: grey for considering the 10-mer at the observed cut only (5' only); orange for SELMA. The published approaches (blue bars in Fig. 2d-e) are not applicable for DNase-seq because they are specifically designed for ATAC-seq. (**e-j**) Pearson correlation coefficient between genome-wide observed (OBS) and bias-expected (EXP) cleavages for ENCODE ATAC-seq data from different tissues. (**k-n**) Comparison between bias scores estimated from ATAC-seq and modified Tn5 based techniques: THS-seq with standard Tn5 (**k**); THS-seq with mutated Tn5 (**l**); fast-ATAC-seq (**m**); and omni-ATAC-seq (**n**).

## Supplementary Figure 4

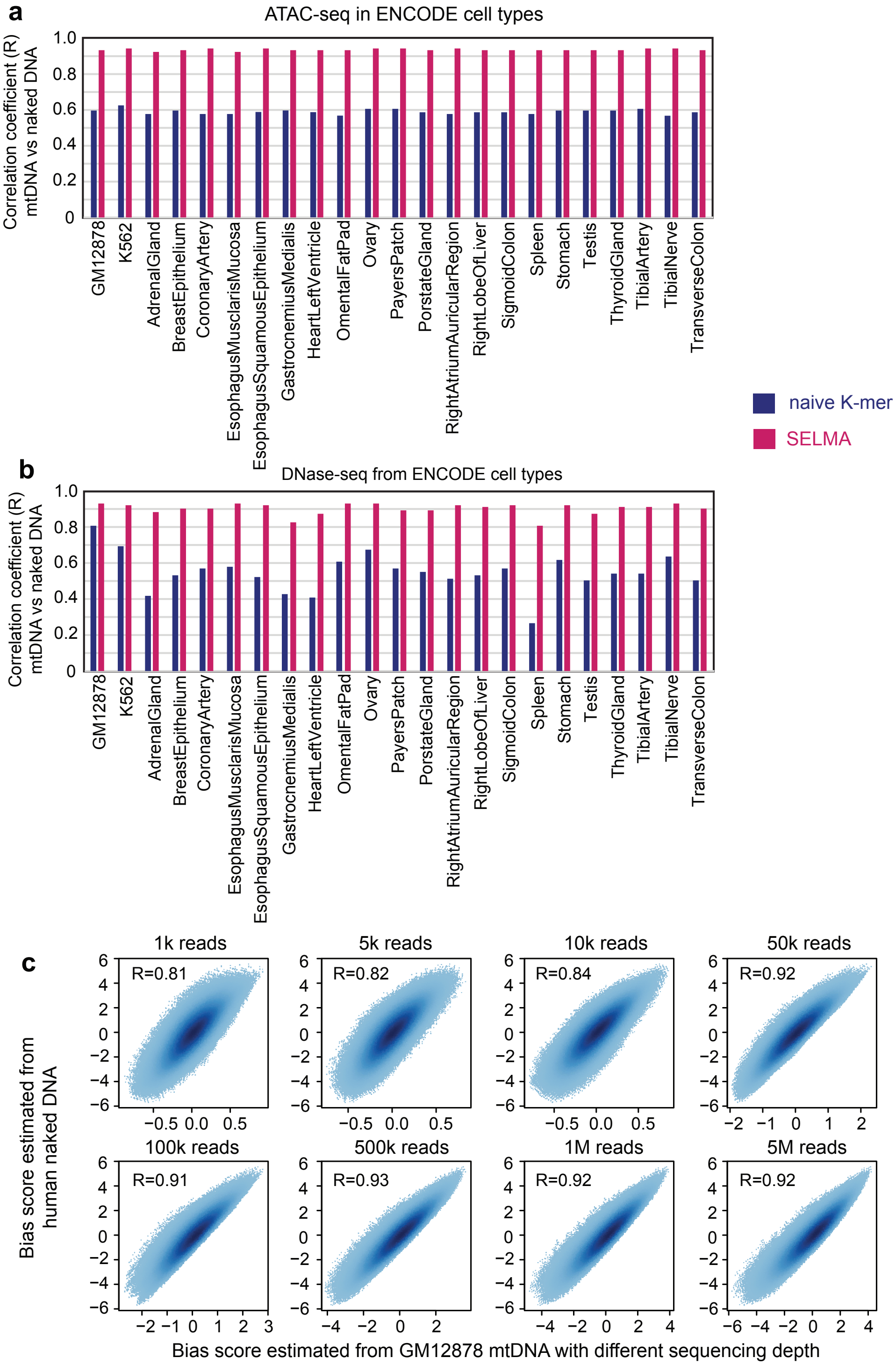


**Supplementary Figure 4. SELMA bias estimation using mtDNA reads.** (**a**) Bar plots demonstrating the Pearson correlation coefficients (y-axis) between 10-mer bias scores estimated from mtDNA (mtDNA reads from chromatin ATAC-seq data generated from ENCODE project in different cell types) and genomic DNA (reads from naked DNA ATAC-seq data), comparing the naïve k-mer model (blue) and SELMA (red). (**b**) Similar to (**a**) but for ENCODE DNase-seq data in the same cell types. (**c**) Scatter plots demonstrating the consistency of 10-mer bias scores estimated from mtDNA reads with different read counts (x-axis) and genomic DNA reads from naked DNA ATAC-seq data (y-axis). The Pearson correlation coefficients were labeled in the top left corner and used in Fig. 3e.

## Supplementary Figure 5

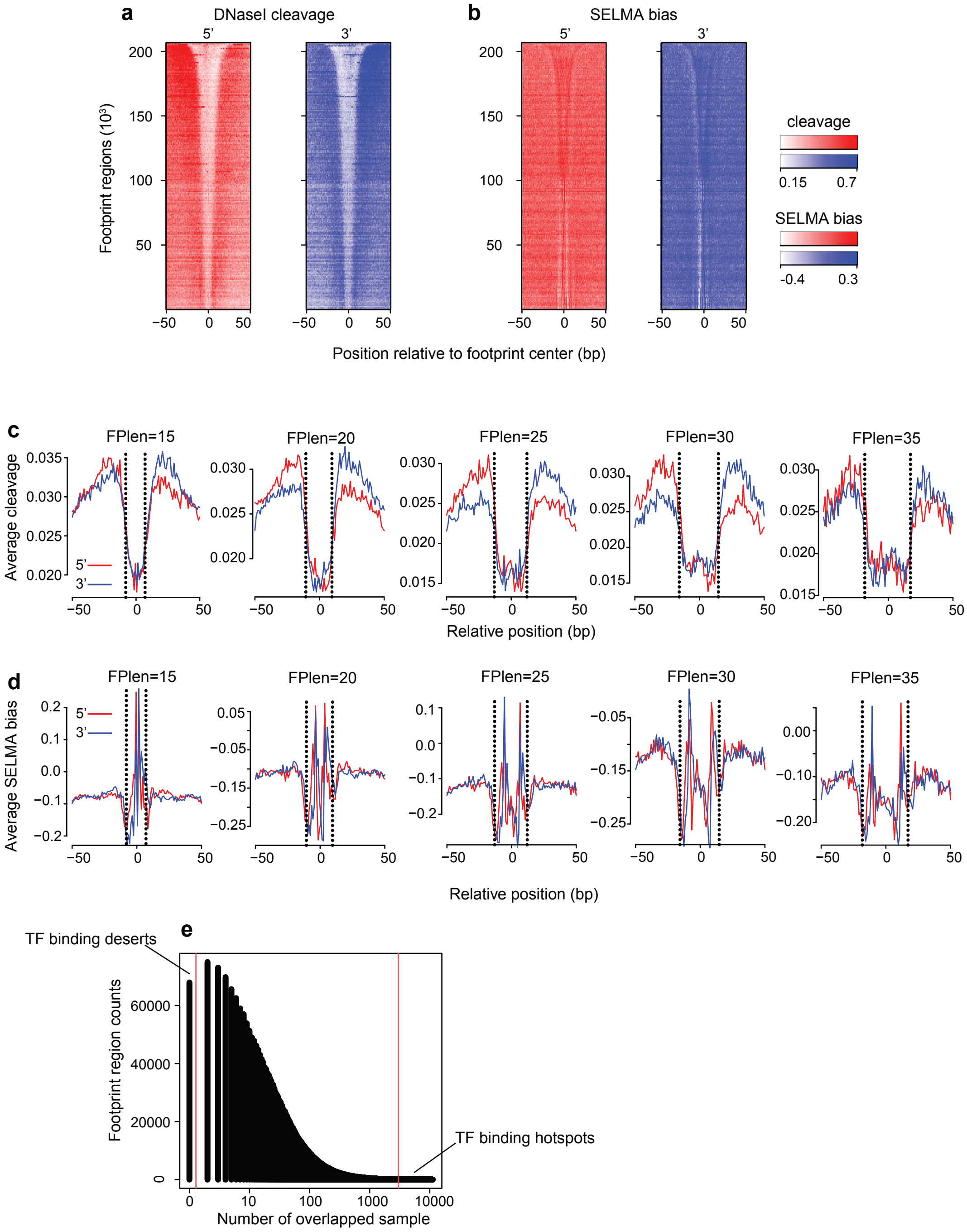


**Supplementary Figure 5. TF binding inference on DNaseI footprint regions is affected by intrinsic cleavage biases and improved by SELMA.** (**a-b**) Heatmaps of DNaseI cleavage patterns in the human K562 cell line (**a**) and SELMA-estimated bias scores (**b**). (**c**) Aggregate plots of DNase-seq cleavage patterns at ENCODE DNaseI footprint regions with different length categories (15, 20, 25, 30, and 35bp). The vertical dotted lines represent the boundaries of the footprint regions. (**d**) similar to (**c**) but for SELMA-estimated bias scores. (**e**) TF binding hotspots and TF binding deserts are defined based on the number of human TF ChIP-seq samples that have peaks overlapping with the footprint regions. The footprint regions overlapping with peaks from >= 3000 ChIP-seq samples are defined as TF binding hotspots; while the footprint regions that do not overlap with any peak from any TF ChIP-seq sample nor any TF motif sites are defined as TF binding deserts.

**Supplementary Figure 6**

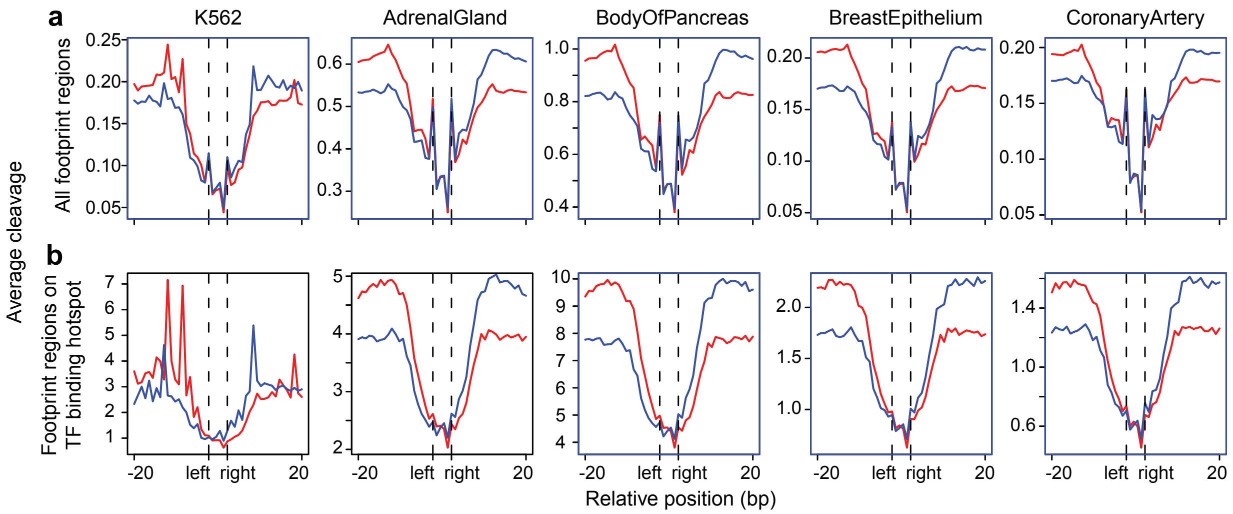


**Supplementary Figure 6. Aggregate plots of DNaseI cleavage patterns of all ENCODE DNaseI consensus footprint regions** (**a**) **and footprint regions overlapping with TF binding hotspots** (**b**). Different columns represent ENCODE DNaseI-seq data from different human cell line (K562) and human tissues.

## Supplementary Figure 7

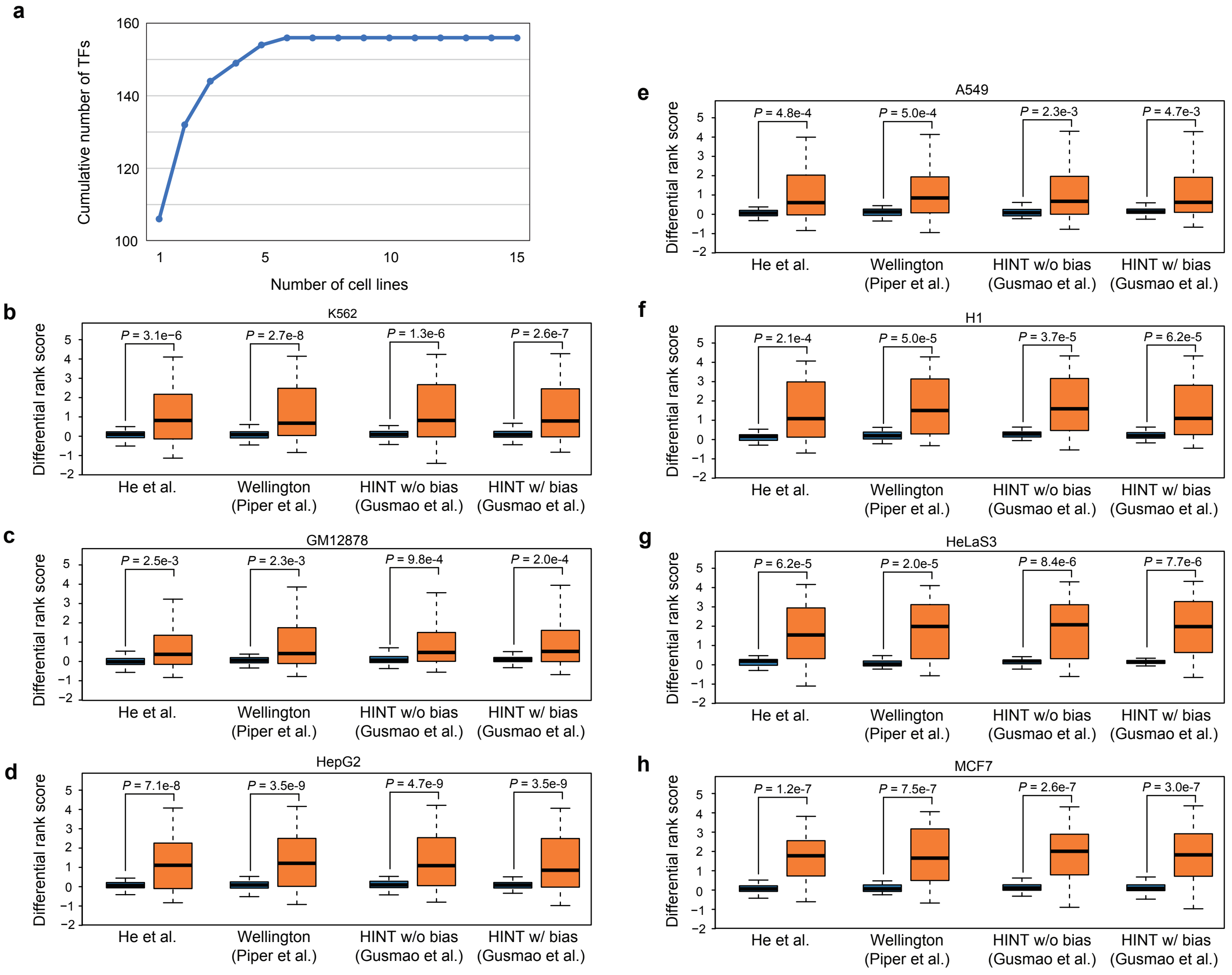


**Supplementary Figure 7. Performance rank scores for transcription factor binding prediction from DNaseI footprint using various methods for different human cell lines.** (**a**) Number of transcription factors (TFs) covered vs. Number of cell lines included in the analysis. Cell lines are ranked by the number of ChIP-seq samples available. (**b-h**) Difference of performance rank scores for TF binding inference from DNaseI footprints using various methods for DNaseI-seq data from different cell lines: (**b**) K562, n = 92 TFs; (**c**) GM12878, n = 53 TFs; (**d**) HepG2, n = 106 TFs; (**e**) A549, n = 31 TFs; (**f**) H1, n = 29 TFs; (**g**) HeLaS3, n = 29 TFs; (**h**) MCF7, n = 35 TFs. Blue, considering random k-mer bias as control; orange, considering SELMA FBS. The centerline, bounds of box, top line, and bottom line of the boxplots represent the median, 25$^{th}$ to 75$^{th}$ percentile range, 25$^{th}$ percentile – 1.5×interquartile range (IQR), and 75$^{th}$ percentile + 1.5×IQR, respectively. P-values are calculated by the one-sided Wilcoxon rank-sum test. The detailed lists of rank scores are available in Supplementary Dataset 2.

## Supplementary Figure 8

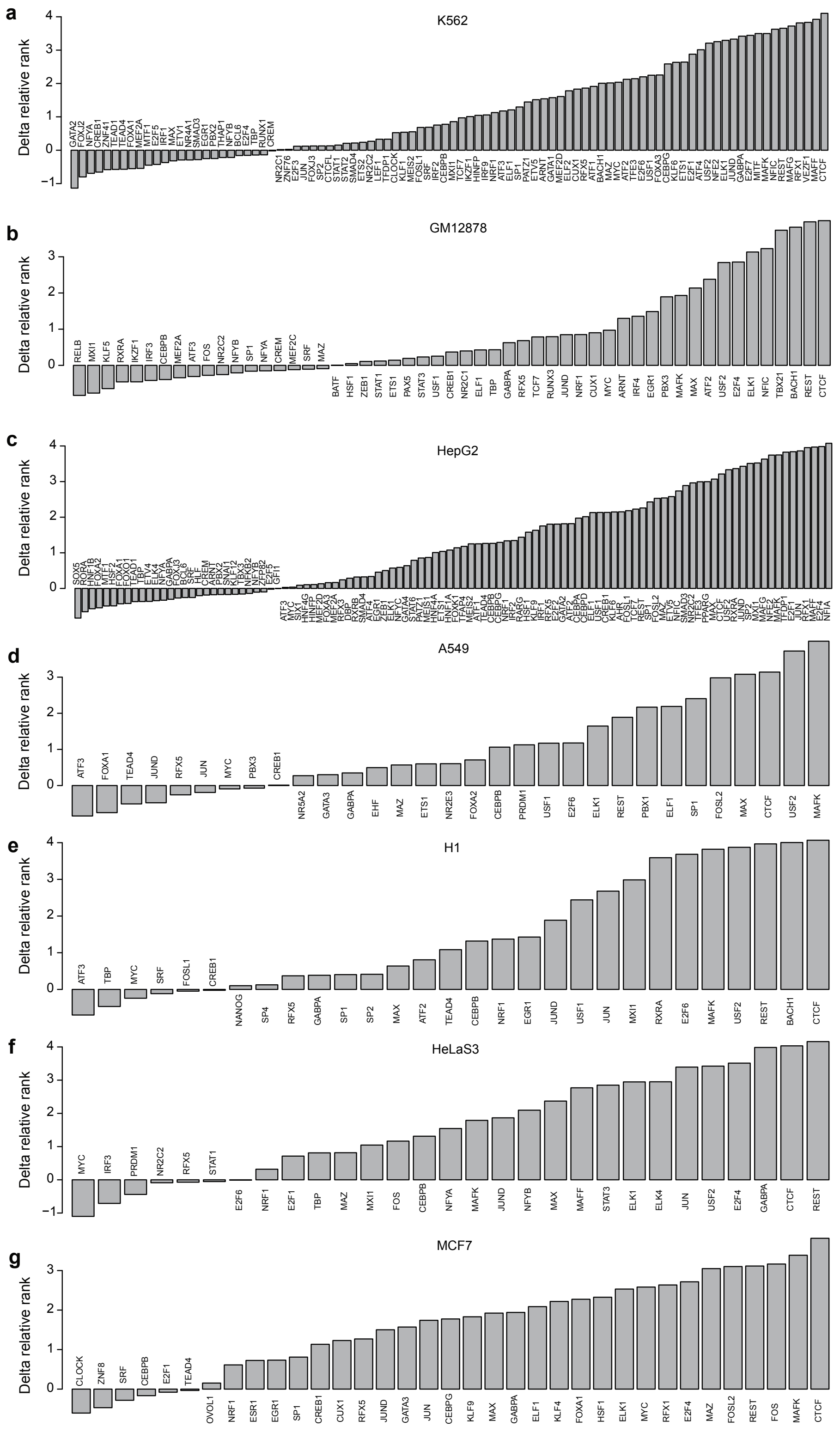

**Supplementary Figure 8. Rank score differences for all TFs in each cell line.** Barplots show the difference in relative rank of TF binding inference from data used in Fig. 4j and Supplementary Fig. 7. Different panels are for TF data in different cell lines: K562 (**a**), GM12878 (**b**), HepG2 (**c**), A549 (**d**), H1 (**e**), HeLaS3 (**f**), and MCF7 (**g**). The complete lists are available in Supplementary Dataset 2.

## Supplementary Figure 9

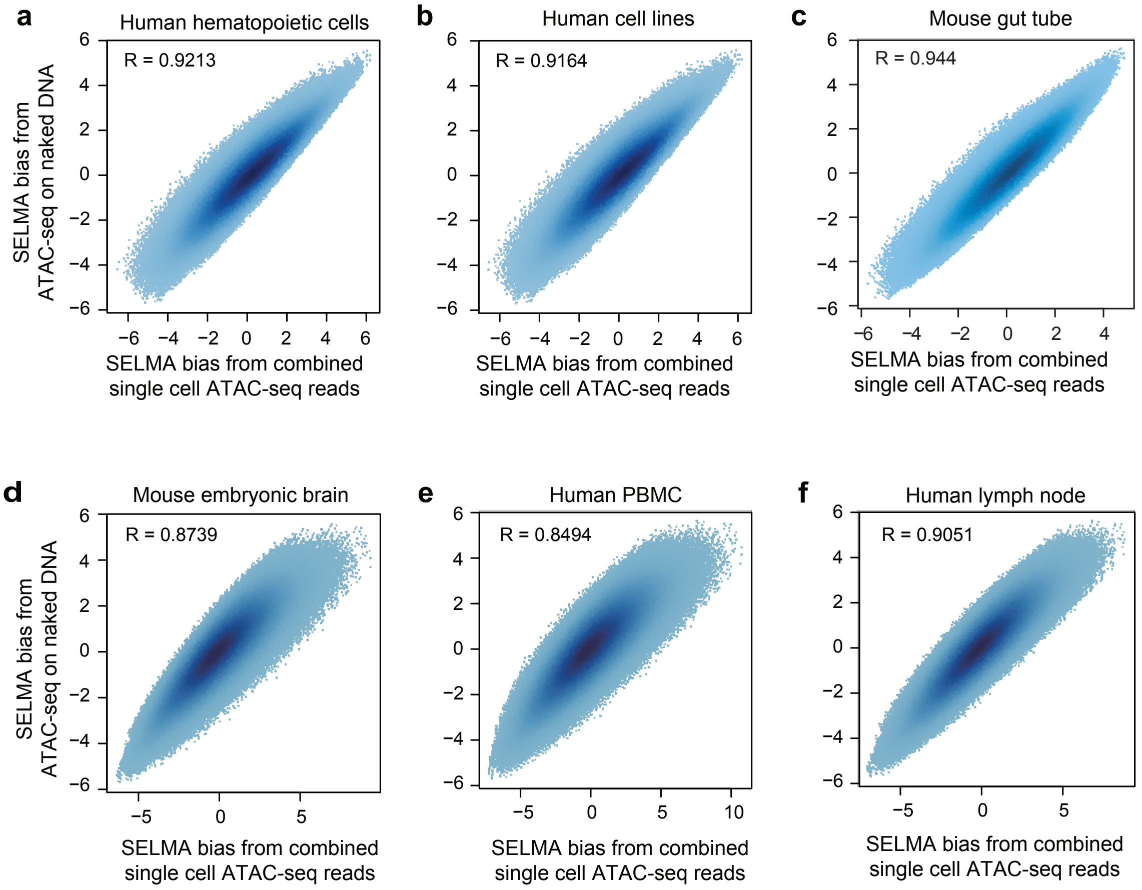


**Supplementary Figure 9. Scatter plots comparing the SELMA bias estimated from bulk naked DNA ATAC-seq data (y-axis) and from different scATAC-seq datasets (x-axis)** for human hematopoietic cells (**a**), mixed human cell lines (**b**), mouse primitive gut tube (**c**), and 10x Single Cell Multiome data for mouse embryonic brain (**d**), human PBMC (**e**), and human lymph node (**f**). The bias scores for (a-c) are calculated using mtDNA reads. The bias scores for (**d**-**f**) were calculated using chromatin reads because the mtDNA reads have been automatically excluded in the fragment data files preprocessed by the 10x Cell Ranger ARC pipeline. R represents the Pearson correlation coefficient.

**Supplementary Figure 10**

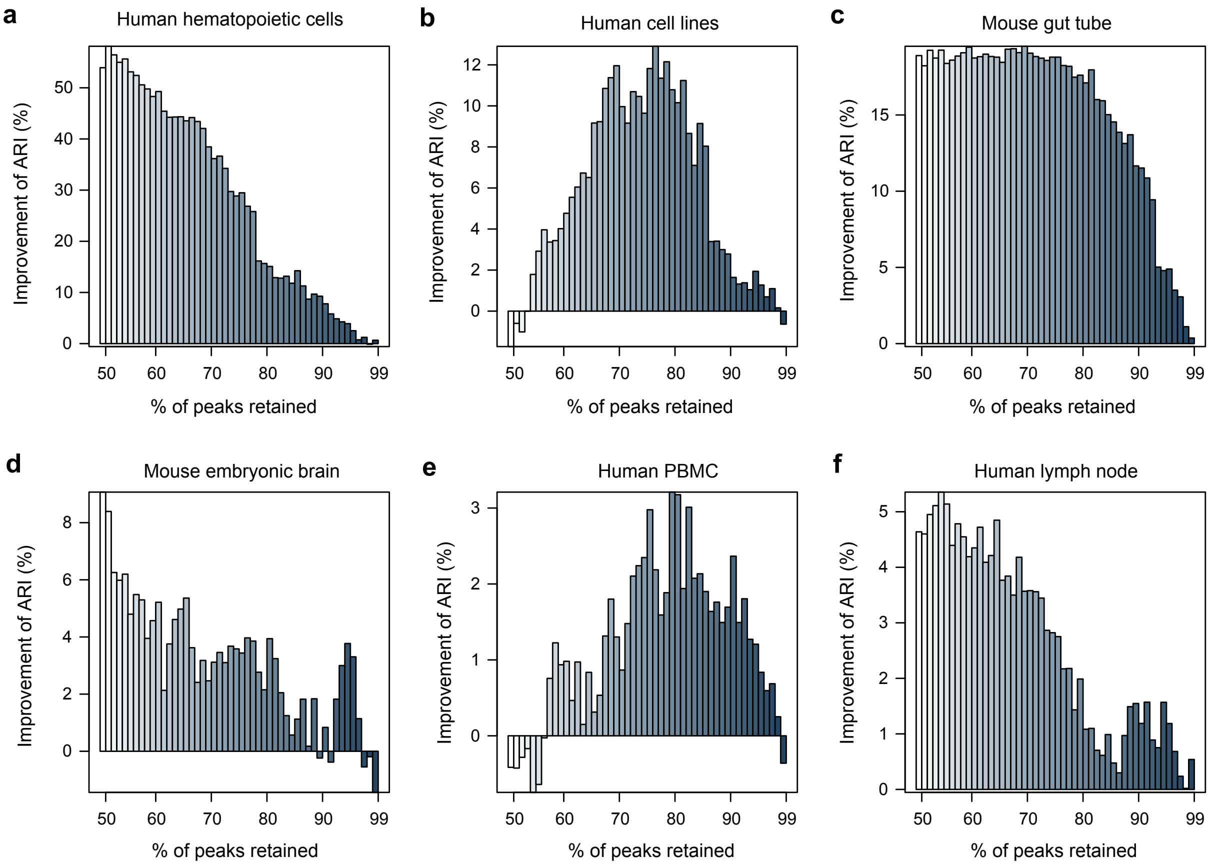


**Supplementary Figure 10. Improvement of cell clustering accuracy after excluding scATAC-seq peaks with high PBS.** Y-axis represents the percentage changes of the adjusted rand index (ARI) between K-means clustering results and the ground truth cell labels. X-axis represents the percent of peaks retained for clustering (50%~99%) after the peaks with highest PBS were removed. Different panels represent data from different biological systems: human hematopoietic cells (**a**), mixed human cell lines (**b**), mouse gut tube (**c**), mouse embryonic brain (**d**), human PBMC (**e**), and human lymph node (**f**).

## Supplementary Figure 11

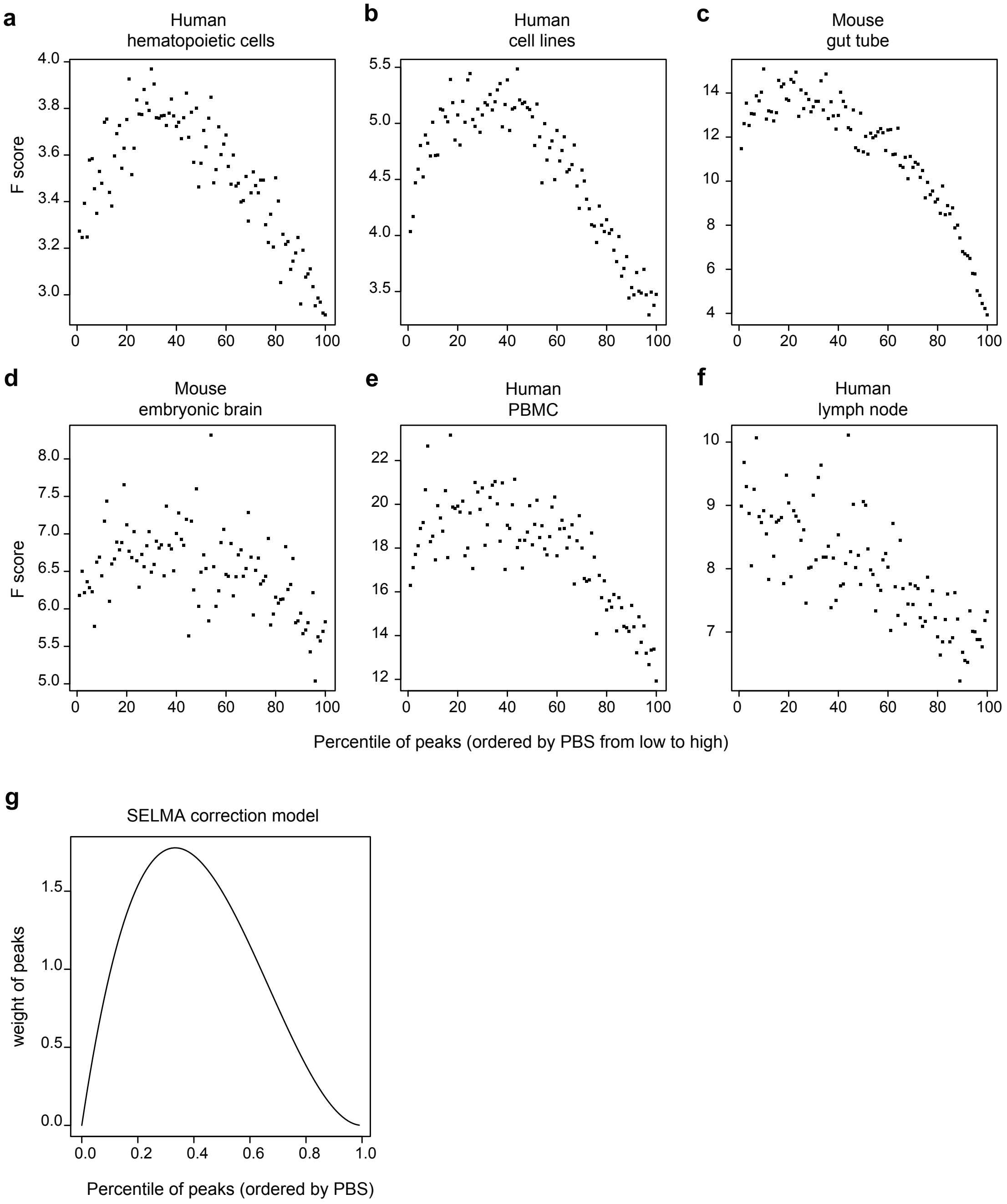


**Supplementary Figure 11. SELMA single-cell peak bias correction model development.** (**a-f**) ANOVA results for predicting cell type labels with the scATAC-seq signals. An F score was calculated for each peak to measure the importance of the peak to cell type classification. Peaks were then ranked by PBS score, and the median F score of the peaks in each PBS percentile were plotted. Different panels represent data from different biological systems: human hematopoietic cells (**a**), mixed human cell lines (**b**), mouse gut tube (**c**), mouse embryonic brain (**d**), human PBMC (**e**), and human lymph node (**f**). (**g**) PBS percentile weight function used in the SELMA single cell peak bias correction model.

## Supplementary Figure 12

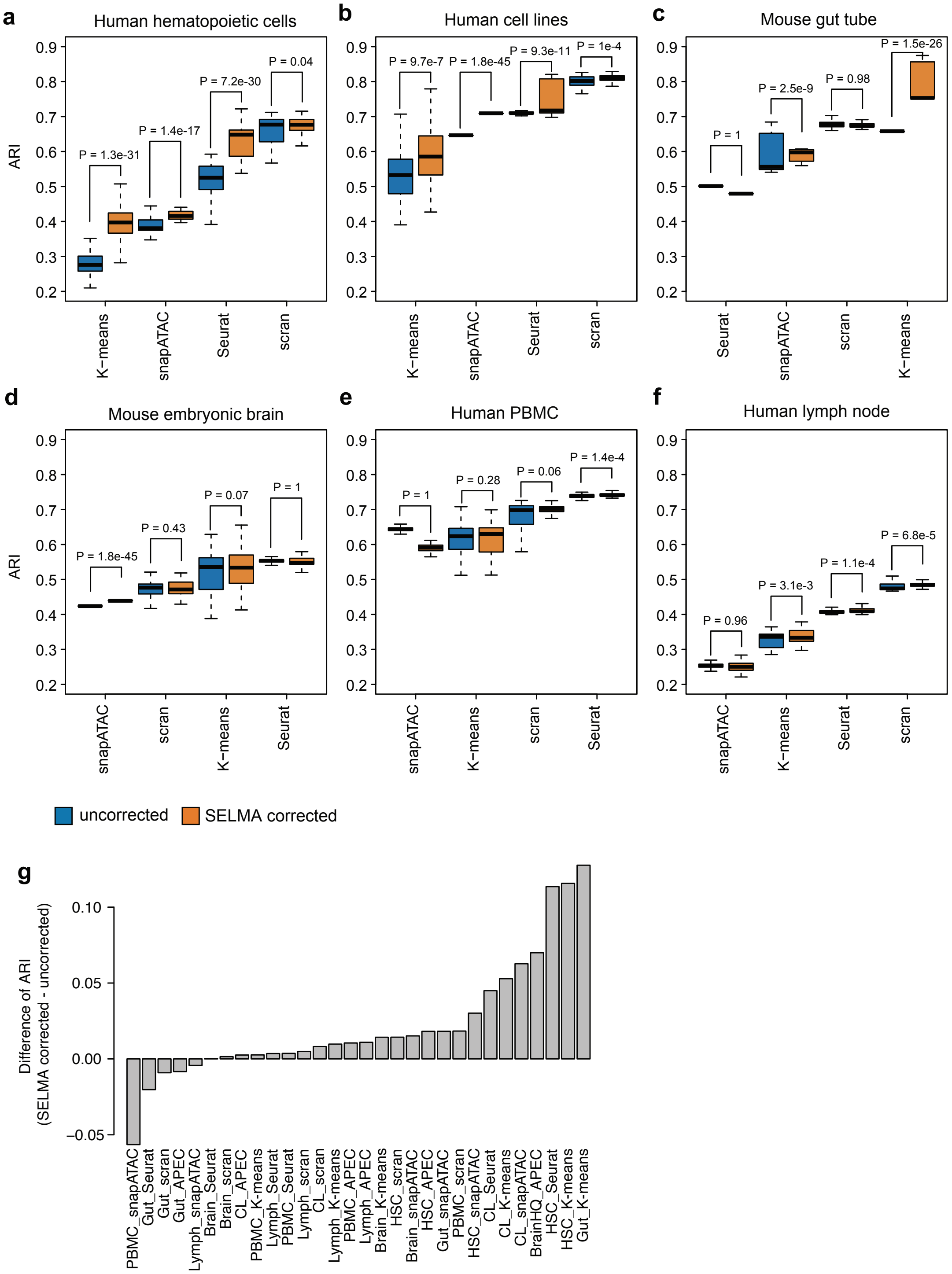


**Supplementary Figure 12. SELMA single-cell peak bias correction model improves single-cell ATAC-seq cell clustering.** (**a-f**) Distributions of ARIs generated from 100 runs with different random seeds, compared between uncorrected raw data (blue) and SELMA-corrected data (orange), for each clustering method in each scATAC-seq sample (n=100 for each boxplot). The centerline, bounds of box, top line, and bottom line of the boxplots represent the median, 25$^{th}$ to 75$^{th}$ percentile range, 25$^{th}$ percentile – 1.5×interquartile range (IQR), and 75$^{th}$ percentile + 1.5×IQR, respectively. P-values were calculated by the one-sided Wilcoxon rank-sum test. (**g**) Difference in ARI after bias correction. HSC: human hematopoietic cells, CL: mixed human cell lines, Gut: mouse gut tube, Brain: mouse embryonic brain, PBMC: human PBMC, Lymph: human lymph node.
