# Supplementary material for: Intrinsic bias estimation for improved analysis of bulk and single-cell chromatin accessibility profiles using SELMA
Source: Nat Commun. 2022 Sep 21;13:5533. doi: 10.1038/s41467-022-33194-z (PMC9492688; doi:10.1038/s41467-022-33194-z)
Supplement: Supplementary file 2 — Reporting Summary [file 41467_2022_33194_MOESM2_ESM.pdf]

nature research

Corresponding author(s): Chongzhi Zang
Last updated by author(s): Sep 6, 2022

Reporting Summary

Nature Research wishes to improve the reproducibility of the work that we publish. This form provides structure for consistency and transparency in reporting. For further information on Nature Research policies, see our Editorial Policies and the Editorial Policy Checklist.

Statistics

For all statistical analyses, confirm that the following items are present in the figure legend, table legend, main text, or Methods section.

n/a	Confirmed	
☐	☒	The exact sample size (n) for each experimental group/condition, given as a discrete number and unit of measurement
☐	☒	A statement on whether measurements were taken from distinct samples or whether the same sample was measured repeatedly
☐	☒	The statistical test(s) used AND whether they are one- or two-sided<br>*Only common tests should be described solely by name; describe more complex techniques in the Methods section.*
☐	☒	A description of all covariates tested
☐	☒	A description of any assumptions or corrections, such as tests of normality and adjustment for multiple comparisons
☐	☒	A full description of the statistical parameters including central tendency (e.g. means) or other basic estimates (e.g. regression coefficient) AND variation (e.g. standard deviation) or associated estimates of uncertainty (e.g. confidence intervals)
☐	☒	For null hypothesis testing, the test statistic (e.g. F, t, r) with confidence intervals, effect sizes, degrees of freedom and P value noted<br>*Give P values as exact values whenever suitable.*
☒	☐	For Bayesian analysis, information on the choice of priors and Markov chain Monte Carlo settings
☒	☐	For hierarchical and complex designs, identification of the appropriate level for tests and full reporting of outcomes
☐	☒	Estimates of effect sizes (e.g. Cohen's d, Pearson's r), indicating how they were calculated

Our web collection on statistics for biologists contains articles on many of the points above.

Software and code

Policy information about availability of computer code

Data collection	Publicly available ATAC-seq, single-cell ATAC-seq, DNase-esq and ChIP-seq data used in this study were collected from Gene Expression Omnibus (GEO), Sequence Read Archive (SRA) and the ENCODE project data portal. The metadata and accession numbers are listed in Supplementary Dataset 1.<br><br>The motifs of human TFs were collected from the HOCOMOCO database36 (v11).<br><br>The genome-wide DNaseI consensus footprint regions were downloaded from (https://resources.altius.org/~jvierstra/projects/footprinting.2020/consensus.index/).
Data analysis	Processing of bulk ATAC-seq and DNase-seq data: Raw sequencing reads were aligned to the GRCh38 (hg38) reference genome with bowtie2 (v2.2.9) (-X 2000 for paired end data). Low-quality reads (MAPQ < 30) were discarded. For paired-end sequencing data, reads with two ends aligned to different chromosomes (chimeric reads) were also discarded. For paired-end data, reads with identical 5' end positions for both ends were regarded as redundant reads and discarded. The nonredundant reads were separated into chromosomal DNA (chromatin reads) and mitochondrial DNA (mtDNA reads) based on their genomic location. Peak detection was performed on the nonredundant chromatin reads using MACS2 (v2.1.2) (-q 0.01, --extsize 50) and +/- 200bp centered on the peak summits was collected as the genome-wide chromatin accessible regions. The accessible regions in each dataset were separated into 5 groups from highest accessibility to lowest accessibility based on the read count on each peak. The 5' end nucleotides of each read were piled up to generate the genome-wide observed cleavage profile.<br><br>Processing of single-cell ATAC-seq data: For the human HSC and cell line sample, raw sequencing reads were aligned to the GRCh38 (hg38) reference genome with bowtie2 (-X 2000). Low quality reads (MAPQ < 30), chimeric reads and duplicate reads for each individual cell were discarded. For the mouse gut tube sample, scATAC-seq data from the 10x Genomics platform were preprocessed with Cell Ranger ATAC (v6.1.1) with the default parameters to generate fragments for each individual cell. The fragment file from the standard Cell Ranger ATAC output was used as input for the subsequent analysis. Because the Cell Ranger ATAC pipeline shifted from the Tn5 cleavage sites to +4/-5 bp in

generating the fragment file, the coordinates were shifted back to represent the actual cleavage loci. For all datasets, cells with more than 10,000 reads were retained for analysis.

TF motif analysis. The genome-wide motif sites of TFs were detected by FIMO (v4.12.0) in the MEME package50. Motif sites located outside of the genome-wide 36bp unique mappable regions were excluded from the analysis. In total, 61,531,309 motif sites for 156 TF motifs from the human genome were included in the analysis.

The observed DNaseI cleavage profile from a DNase-seq dataset and DNaseI SELMA bias scores across +/- 50bp centered on the footprint region were plotted as heatmaps (Fig. 4a,b). The footprint regions were ordered by the footprint lengths, and each 1000 footprint regions with similar lengths were compressed as one row in the heatmap for better visualization. The +/- strand signals were plotted separately. We aligned the footprint regions based on the two observed bias spikes in each footprint region (located 7bp to the right of the left boundary and 7bp to the left of the right boundary of the footprint, labeled as -0 and +0 in Fig. 4c-h). The center regions between the bias spikes were scaled to 4 bins to align footprint regions with different lengths.

For manuscripts utilizing custom algorithms or software that are central to the research but not yet described in published literature, software must be made available to editors and reviewers. We strongly encourage code deposition in a community repository (e.g. GitHub). See the Nature Research guidelines for submitting code & software for further information.

Data

Policy information about availability of data

All manuscripts must include a data availability statement. This statement should provide the following information, where applicable:

- Accession codes, unique identifiers, or web links for publicly available datasets
- A list of figures that have associated raw data
- A description of any restrictions on data availability

The mouse gut tube scATAC-seq dataset is available in the Gene Expression Omnibus (GEO) with accession number GSE168373 (https://www.ncbi.nlm.nih.gov/geo/query/acc.cgi?acc=GSE168373). The 10x Single Cell Multiome datasets are downloaded from 10x Genomics website (https://www.10xgenomics.com/resources/datasets/fresh-embryonic-e-18-mouse-brain-5-k-1-standard-2-0-0, https://www.10xgenomics.com/resources/datasets/pbmc-from-a-healthy-donor-no-cell-sorting-10-k-1-standard-2-0-0, https://www.10xgenomics.com/resources/datasets/fresh-frozen-lymph-node-with-b-cell-lymphoma-14-k-sorted-nuclei-1-standard-2-0-0). All publicly available data used in this study are downloaded from the GEO or the ENCODE project data portal. Accession numbers for all the GEO and ENCODE data used in the study are available in Supplementary Dataset 1. The human (hg38) and mouse (mm10) reference genome information was downloaded from UCSC genome browser (https://hgdownload.soe.ucsc.edu/downloads.html).

Field-specific reporting

Please select the one below that is the best fit for your research. If you are not sure, read the appropriate sections before making your selection.

[x] Life sciences   [ ] Behavioural & social sciences   [ ] Ecological, evolutionary & environmental sciences

For a reference copy of the document with all sections, see nature.com/documents/nr-reporting-summary-flat.pdf

Life sciences study design

All studies must disclose on these points even when the disclosure is negative.

Sample size	Public data used in this study were collected exhaustively. Analyses were performed on all available and applicable data collected and represent the population. No sample size calculation was performed.
Data exclusions	In single-cell ATAC-seq data analysis, cells with low quality data were discarded following the criteria: For scATAC-seq datasets, cells with less than 10,000 reads were discarded. For 10x Single Cell Multiome datasets, cells were pre-selected by Cell Ranger ARC. Cells and with less than 10,000 reads in either scRNA-seq or scATAC-seq components were discarded. Cell types represented by fewer than 10 cells were discarded. Potential accessible regions with fewer than 10 covered reads or more than 4,000 covered reads were discarded in the analysis.
Replication	Public data used in this study were collected exhaustively. All conclusions made in this study were reproducible in multiple biological systems based on publicly available data collected. In single-cell ATAC-seq data analysis, available data from all batches and replicates were used in the analyses. In TF binding inference, we collected as many public TF ChIP-seq datasets as possible for QC, and used one sample for each TF in each cell type for a fair systematic comparison.
Randomization	1) Randomization was used to generate negative control in TF binding inference from footprints (Fig. 4j). A random k-mer bias was generated by random permutation of the SELMA k-mer bias table to calculate the randomized FBS for each footprint region. To calculate the random k-mer bias, we randomly permuted the SELMA k-mer bias table and generated the k-mer bias table for a “simulated enzyme”. We used this “simulated” bias table to calculate the FBS for each footprint region and performed TF inference modeling similar to what we did for the DNaseI SELMA FBS. This permutation was performed 100 times to generate 100 performance rank scores for random k-mer bias used as controls.<br><br>2) Randomization was used to generate negative control for the high-PBS peak removal approach for single-cell ATAC-seq clustering (Fig. 6a-f). At each percentage of peak removal, we randomly selected the same percentage of peaks as a control to estimate the relative rank of the clustering performance from using all peaks and using retained peaks. For each percentage from 50% to 99%, we randomly sampled peaks 100 times and defined the relative rank as the number of ARIs from random samples that were less than the ARI from the same percentage of retained peaks or all peaks.

	3) Randomization was used to generate different initialization seeds for single-cell ATAC-seq clustering analysis. To evaluate the robustness of clustering results for each method, we repeated the clustering for 100 times with different random seeds, and used the average and standard deviation of the ARIs from the 100 runs as the evaluation metrics.
Blinding	Blinding was not applicable to this study. All analyses were performed on publicly available data with rigorous statistical assessments.

Reporting for specific materials, systems and methods

We require information from authors about some types of materials, experimental systems and methods used in many studies. Here, indicate whether each material, system or method listed is relevant to your study. If you are not sure if a list item applies to your research, read the appropriate section before selecting a response.

Materials & experimental systems

n/a	Involved in the study
☒	☐ Antibodies
☒	☐ Eukaryotic cell lines
☒	☐ Palaeontology and archaeology
☒	☐ Animals and other organisms
☒	☐ Human research participants
☒	☐ Clinical data
☒	☐ Dual use research of concern

Methods

n/a	Involved in the study
☒	☐ ChIP-seq
☒	☐ Flow cytometry
☒	☐ MRI-based neuroimaging
