# Supplementary material for: Intrinsic bias estimation for improved analysis of bulk and single-cell chromatin accessibility profiles using SELMA
Source: Nat Commun. 2022 Sep 21;13:5533. doi: 10.1038/s41467-022-33194-z (PMC9492688; doi:10.1038/s41467-022-33194-z)
Supplement: Supplementary file 3 — Description of Additional Supplementary Files [file 41467_2022_33194_MOESM3_ESM.pdf]

Title: Supplementary Dataset 1:
Description: Datasets used in the study. Each dataset includes name, experimental type, species and genome version, source database, and accession number.

Title: Supplementary Dataset 2:
Description: TF inference performance improvement measured by differential relative ranks for all TF samples. These are the original data for plotting Figures 4j, S7, and S8. Results from each footprint score calculation method are shown in a separate sheet: (a) He et al.; (b) Wellington; (c) HINT without bias; (d) HINT with bias.

Title: Supplementary Dataset 3:
Description: Fraction of motif-containing sites with TF occupancy, for sites with highFBS footprints and sites with low-FBS footprints, and their log2 ratio. These are the original data for plotting Figure 4k.

Title: Supplementary Dataset 4:
Description: Cell numbers for each cell type/batch/cluster in each single-cell ATACseq sample shown in the boxplots in Figure 5c, f, i, l, o, r.

Title: Supplementary Dataset 5:
Description: Performance of scATAC-seq cell clustering using uncorrected data and using SELMA model-corrected data, measured by adjusted Rand index (ARI). These are the original data for plotting Figure 6h-m.
